# Tension Subdural Hygroma Following Resection of Posterior Fossa Tumour in a Child: A new clinico-radio-pathological entity

**DOI:** 10.18295/squmj.3.2024.015

**Published:** 2024-05-27

**Authors:** Mahesh K. Pillai, Rajeev Kariyattil, Rajesh Chhabra, Venkatesh Govindaraju, Koshy K. Kottoorazhikam

**Affiliations:** 1 Department of Neurosurgery, Sultan Qaboos University Hospital, Sultan Qaboos University, Muscat, Oman; 2 Department of Neurosurgery, Postgraduate Institute of Medical Education and Research, Chandigarh, India

**Keywords:** Child, Posterior Fossa Tumour, Postoperative Period, Hydrocephalus, Subdural Hygroma

## Abstract

Persistent hydrocephalus is common in children after resection of posterior fossa tumours. However, occurrence of subdural hygroma is very rare. We report the case of a 14-month-old child who presented at a paediatric neurology clinic in Muscat, Oman in 2021 who developed a tense subdural hygroma with stable hydrocephalus, in the early postoperative period, following posterior fossa tumour resection. We describe the distinctive clinical, radiological and pathological features associated with the development of a tense subdural hygroma. We also discuss the management by cerebrospinal fluid diversion, which includes either a ventriculoperitoneal or subduroperitoneal shunt. This unique condition is distinguished from external hydrocephalus by features that are critical to the management strategy.

The incidence of non-communicating hydrocephalus (HC) in children with posterior fossa tumour (PFT) is 70–90%.^1^ HC will persist in approximately 30% of them after resection of the tumour and may have a communicating component to it.^2^–^6^ No report exists, to date, that discusses the development of tense pseudomeningocele (PMC) due to tense subdural and interhemispheric cerebrospinal fluid (CSF) collection associated with regression of HC after PFT resection. We report such a case and introduce the term ‘tension subdural hygroma’ to describe this rare, but distinct clinical, radiological and pathological phenomenon.

## Case Report

**Figure 1:**
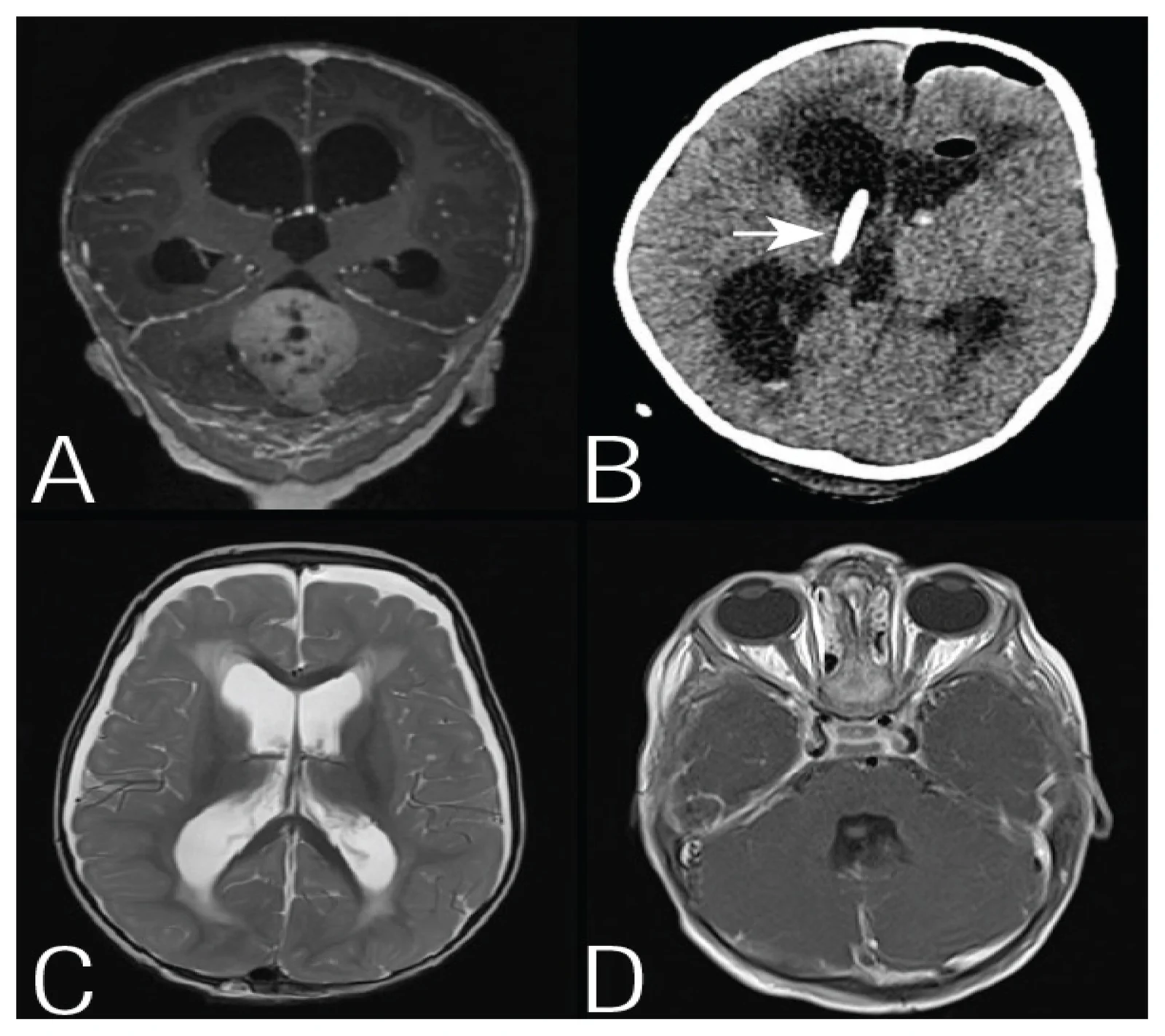
**A:** A coronal T1W, gadolinium enhanced magnetic resonance imaging (MRI) scan of the brain showing midline posterior fossa tumour with hydrocephalus. **B:** An axial section of a postoperative computed tomography scan 24 hours after clamping the external ventricular drain showing persistent hydrocephalus and ventricular catheter in the right lateral ventricle (arrow). **C:** An axial T2W postoperative MRI of the brain 72 hours after removal of external ventricular drain showing thin subdural hygroma and regression of hydrocephalus. D: An axial T1W, gadolinium enhanced MRI showing no residual tumour.

A 14-month-old male child presented to a paediatric neurology clinic in Muscat, Oman in 2021 with vomiting, irritability and imbalance while sitting/walking since 2–3 weeks. Clinically, the patient was awake and alert, but was irritable and had truncal ataxia. Pupils were equal and reacting to light and fundi showed no papilledema. Magnetic resonance imaging (MRI) of brain showed a large midline PFT, the features of which were suggestive of medulloblastoma. There was associated HC with periventricular lucency [Figure 1A]. He underwent a modified telo-velar approach and gross total excision of the tumour. An external ventricular drain (EVD) was placed through a right sided Frazier’s burr hole, immediately before surgery. The EVD was kept clamped postoperatively. After 24 hours, the postoperative computerised tomography (CT) scan showed a clear tumour bed, some blood in the frontal horns of lateral ventricles and the third ventricle. The cerebral aqueduct was patent. The HC was persisting with the Evans index remaining the same as in the preoperative scan [Figure 1B]. EVD was removed since there was no worsening of the HC after clamping the drain for 24 hours. An MRI scan of the brain and spine on the third postoperative day showed a new bilaterally symmetrical subdural hygroma and reduction in HC [Figure 1C]. There was no residual tumour or spine metastasis [Figure 1D].

**Figure 2:**
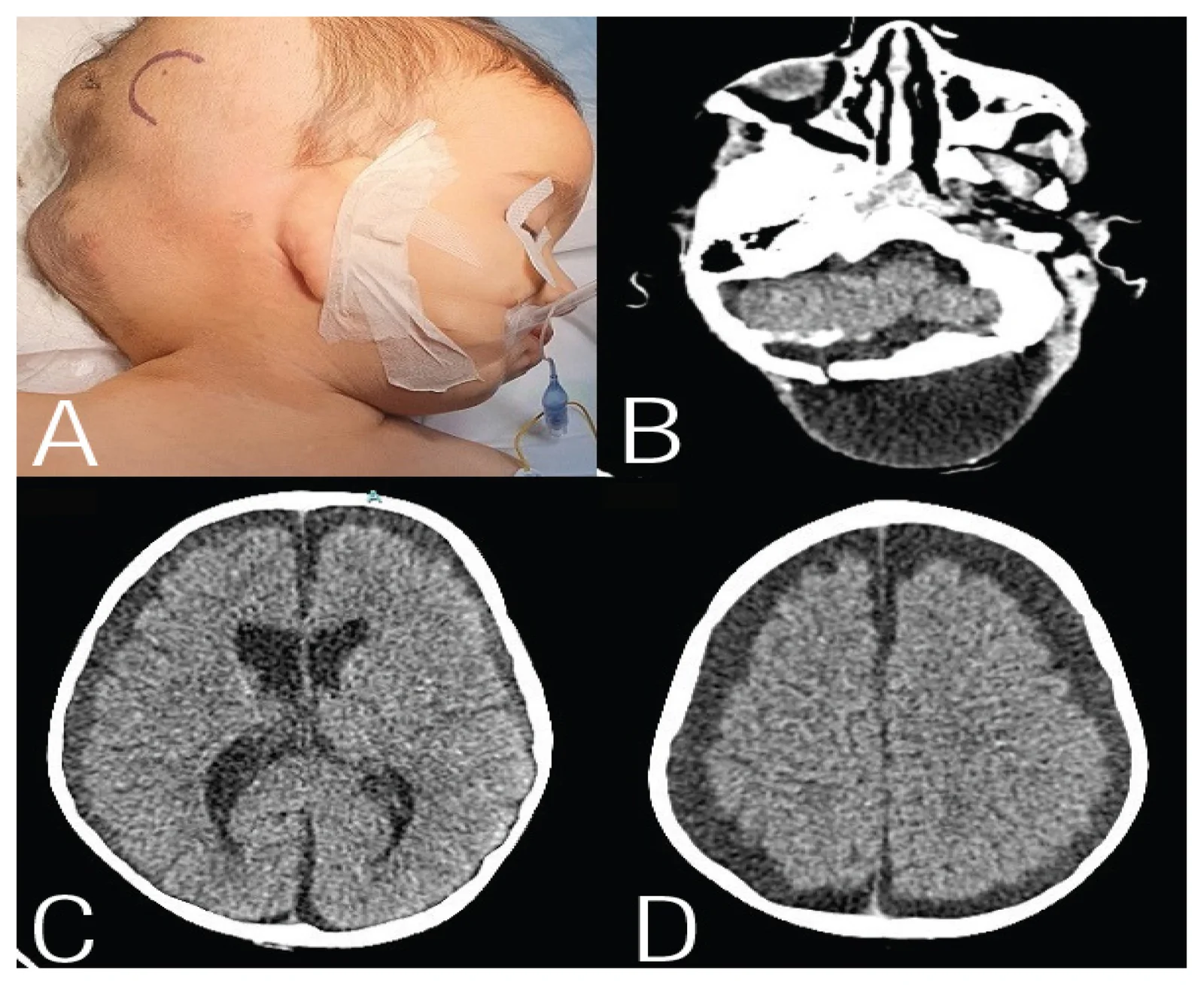
**A:** Photograph showing a tense pseudomeningocele at the external ventricular drain site (arrow down) and surgical site (arrow up) immediately before shunt surgery as well as the shunt incision mark (asterisk). **B:** An axial view of a computed tomography (CT) scan of the brain showing the pseudomeningocele (asterisk). **C & D:** A CT of the brain showing the progression of subdural hygroma and further regression of hydrocephalus.

The child then developed a PMC at the surgical and EVD site on the fifth postoperative day. The PMC progressed despite drainage of CSF via lumbar puncture on the 5^th^ and 12^th^ postoperative days. By the fourteenth day the PMC was tense [Figure 2A]. The child was afebrile but irritable and oral intake was poor with episodes of vomiting. A CT scan of the brain showed very large hypodense collections at the surgical site and bilateral convexity and interhemispheric subdural space with reduction of HC [Figure 2B–D]. Analysis of the CSF obtained during lumbar puncture did not show any evidence of infection. He underwent emergency CSF diversion using a medium pressure shunt system. Peroperatively, upon nicking the dura to insert the ventricular catheter, the CSF in the subdural space, under high pressure, jetted out. The catheter had to be advanced for 4–5 cm in an attempt to enter the ventricle and obtain continuous drainage of CSF.

**Figure 3:**
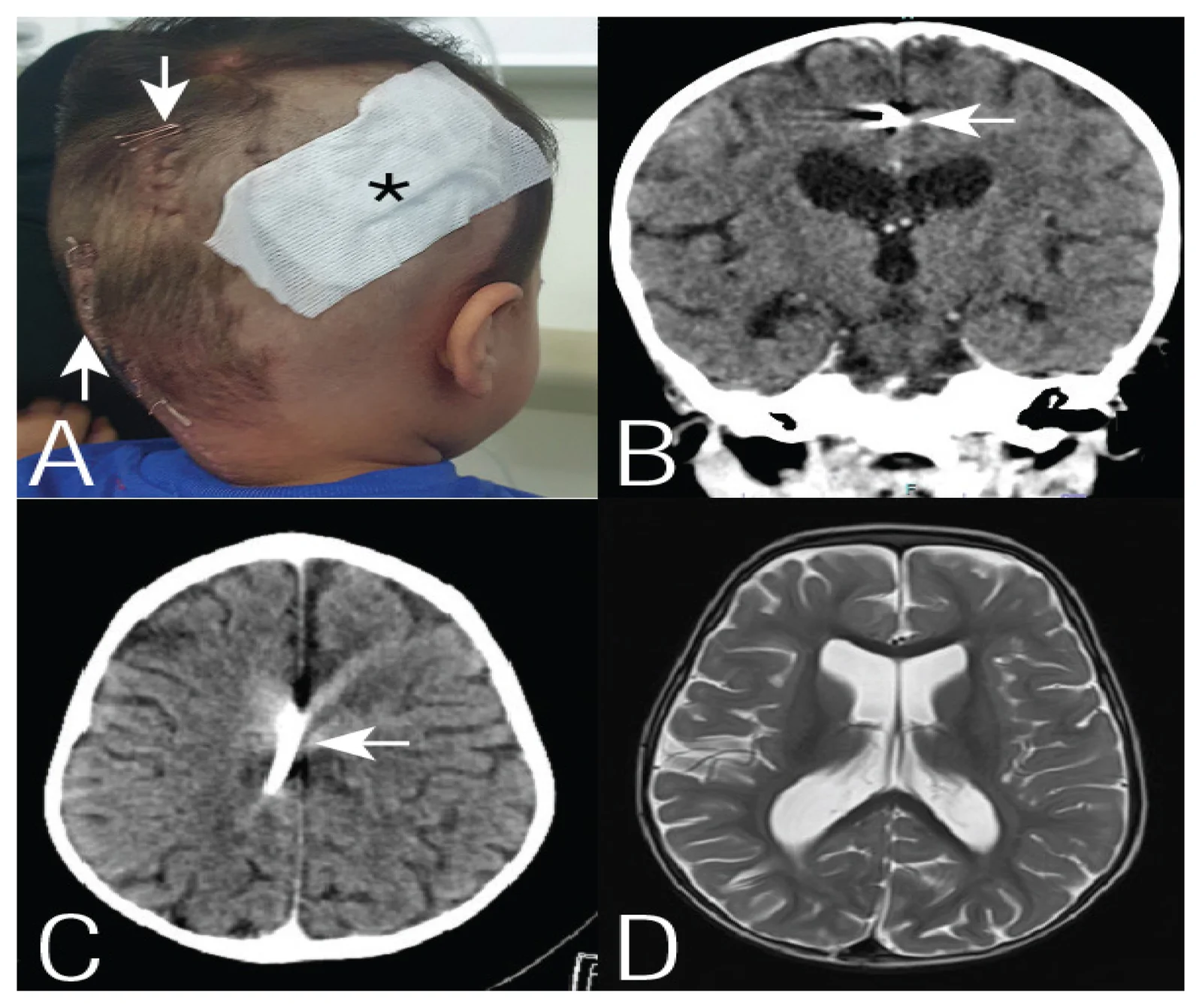
**A:** A photograph showing complete resolution of pseudomeningocele 24 hours after shunt (arrows) and the dressing over shunt insertion site (asterisk). **B & C:** Coronal and axial views of a computed tomography scan of the brain after one month showing complete resolution of the subdural hygroma, regression of the hydrocephalus and shunt tube tip in the interhemispheric subdural space (arrows). **D:** A T2W axial view of a magnetic resonance imaging of the brain after 5 months showing complete resolution of the hydrocephalus and subdural hygroma.

Postoperatively, the PMC resolved completely within a day and the child improved clinically [Figure 3A]. A follow-up CT scan of the brain after one month showed malposition of distal end of the catheter in the interhemispheric subdural space, a stable HC with no periventricular lucency and complete resolution of the subdural collection and PMC, suggestive of a functioning shunt system [Figure 3B–D]. Though a ventriculoperitoneal shunt (VPS) was planned, it a subdural-peritoneal shunt was used, instead, by default. Histopathology examination confirmed the diagnosis of medulloblastoma with extensive nodularity. The child was subsequently transferred to the Department of Oncology for further management. A follow-up MRI of the brain after 6 months showed complete resolution of the HC and subdural hygroma The child has been under regular surveillance since then. Follow-up imaging of the brain, spine and abdomen did not show evidence of recurrence of the tumour, spine metastasis or CSF seeding of tumour in the peritoneal cavity. Intra-thecal chemotherapy was not given since there was no spine metastasis.

Parental consent was obtained for the publication of this case report with photographs and radiological images.

## Discussion

In children with PFT the factors leading to persistence of HC, after tumour resection includes an age of <3 years, duration of illness of <3 months, midline location of the tumour, subtotal resection, preoperative EVD placement, prolonged EVD requirement, early PMC formation, postoperative CSF leak, medulloblastoma/ependymoma histology and greater ventricular index on presentation.^4^^,^^5^^,^^7^–^11^ In this case, the patient underwent right sided EVD immediately before resection of the tumour. In 5 days, the patient developed PMC at the surgical and EVD site, which was progressing to a large and tense PMC, despite 2 attempts of drainage of CSF. The MRI and CT scans (on the 3^rd^ and 14^th^ postoperative days) showed progression of the subdural hygroma and PMC, as well as regression of the HC. The mechanism of development of postoperative subdural hygroma is still a topic of debate. A possible explanation could be that the CSF from the ventricles tracked into the subdural space via the iatrogenic communication created by the EVD and modified telo-velar approach. The progressive egress of CSF, compounded by the communicating nature of HC, led to an increase in subdural hygroma, in terms of both volume and pressure. This in turn led to the development of PMC. The CSF in the subdural space was under high pressure. This was unlike in a subdural hygroma caused by loss of cerebral volume and subdural hygroma due to other causes where there was no direct communication subdural space and the ventricles. This was considered as a separate entity and was termed ‘tension subdural hygroma’. One possible explanation of missing the ventricle while performing VPS was the transient change in the configuration of the right cerebral hemisphere and lateral shift of the right lateral ventricle. This could be due to sudden egress of CSF from the subdural space upon opening the dura. Use of intra-operative image guidance could have avoided the malposition of ventricular catheter. The complete resolution of PMC, subdural hygroma and HC as seen in the post shunt imaging of the brain confirms the dynamic communication between all the three CSF compartments (ventricles, PMC, subdural space) in this child. Other management options were a VPS, burr hole and drainage of subdural hygroma or endoscopic third ventriculostomy. VPS would have resulted in resolution of the subdural hygroma, PMC and HC. Burr hole drainage would have certainly resulted in re-accumulation of the subdural hygroma as evidenced by recurrence of PMC after drainage via lumbar puncture. Endoscopic third ventriculostomy would have been a failure due to the communicating nature of the HC in this case. The researchers compiled a flow chart covering all the possibilities for management [Supplementary Figure 1].

A literature review showed 4 articles reporting development of subdural hygroma following tumour resection, 1 being supratentorial and the other 3 being infratentorial tumors.^12^–^15^ A case report by Behera *et al*., closely resembles the current case.^15^ The authors termed the subdural hygroma as ‘periencephalic subdural panhygroma’. This case did not have a PMC, possibly due to lack of enough tension in the subdural hygroma to produce a PMC. Other possibility was water tight closure of the dura and replacement of the bone flap. The HC also did not regress with the development of subdural hygroma. Moreover the subdural hygroma did not resolve completely after VPS, as evidenced by the postoperative CT scan.^15^ Eguchi *et al*. reported 3 paediatric cases, with tumour in the supratentorial region.^12^ The authors reported these cases as postoperative extra axial CSF collections, irrespective of whether the collection was in the subarachnoid or subdural space. Oomman *et al*. reported cases of two adults with posterior fossa tumour who developed postoperative subdural hygroma (one being a intra axial metastatic cerebellar lesion and the other an intra-fourth ventricular lesion).^13^ These collections were asymmetrical and did not resolve completely after VPS, unlike subdural hygroma. Stavrinos *et al*. reported another case in an adult who developed subdural hygroma following excision of intra axial cerebellar mass.^14^ The subdural hygroma was symmetrical and was managed by burr hole drainage of the supratentorial subdural hygroma and aspiration of the PMC. The other differential diagnosis was external hydrocephalus, where the CSF accumulates in the subarachnoid space. The visualisation of subdural bridging veins over the convexity and absence of widening of cortical sulci in the CT of the brain excluded the possibility of external hydrocephalus.

## Conclusion

This is the second reported case of subdural hygroma following posterior fossa tumour resection in a child. The hallmark features which make it distinct include a tense PMC, progression of subdural hygroma with regression of the HC, CSF in the subdural space under high pressure and CSF diversion resulting in complete resolution of the PMC, subdural hygroma and HC. We introduce the term ‘tension subdural hygroma’ to name this distinct condition. A subdural-peritoneal shunt may be the procedure of choice compared to VPS, because of the easy access of the shunt tube to the convexity of the subdural space compared to ventricles. Further studies and similar case reports are warranted to establish this entity.

## Supplementary Information
